# HDAC6 is a biomarker of leukaemic dissemination and relapse in B-ALL

**DOI:** 10.1038/s41416-026-03539-2

**Published:** 2026-07-26

**Authors:** Karina E. Jiménez-Camacho, Hilda Vargas-Robles, Joseph Adomako, Ana Beatriz Sánchez-Argáez, M. Victor M. Correa-Lara, Antonio Sandoval-Cabrera, Elisa Dorantes-Acosta, Juan Manuel Mejía-Aranguré, Sara Castañeda-Contreras, Juan Carlos Núñez-Enríquez, Minerva Mata-Rocha, Rosana Pelayo, Michael Schnoor

**Affiliations:** 1 Department of Molecular Biomedicine, CINVESTAV, Mexico-City, Mexico; 2 Division of Experimental Hematology and Cancer Biology, Cincinnati Children’s Hospital Medical Center, Cincinnati, OH USA; 3 Advanced Leukemia Therapies and Research Center, Cincinnati Children’s Hospital, Cincinnati, OH USA; 4 Hospital para el Niño, Instituto Materno Infantil del Estado de México, Toluca, Mexico; 5 Facultad de Medicina, Universidad Autónoma del Estado de, Mexico, Mexico; 6 Leukemia Research Biobank, Hospital Infantil de Mexico Federico Gómez, Mexico City, Mexico; 7 Laboratorio de Genómica Funcional del Cáncer, Instituto Nacional de Medicina Genómica (INMEGEN), Mexico City, Mexico; 8 Facultad de Medicina, Universidad Nacional Autónoma de México (UNAM), Mexico City, Mexico; 9 Centro Médico Nacional “La Raza”, Instituto Mexicano del Seguro Social (IMSS), Mexico City, Mexico; 10 Division of Health Research, UMAE Hospital de Pediatría, Centro Médico Nacional Siglo XXI, Instituto Mexicano del Seguro Social, Mexico City, Mexico; 11 Unidad de Investigación Médica en Genética Humana, Unidad Médica de Alta Especialidad, Hospital de Pediatría “Dr. Silvestre Frenk Freund”, Centro Médico Nacional Siglo XXI, IMSS, Mexico City, Mexico; 12 Laboratorio de Oncoinmunología y Citómica, Centro de Investigación Biomédica de Oriente, Instituto Mexicano del Seguro Social Delegación Puebla, Puebla, Mexico

**Keywords:** Acute lymphocytic leukaemia, Prognostic markers

## Abstract

**Background/Objectives:**

Relapse remains the leading cause of treatment failure in B-cell acute lymphoblastic leukaemia (B-ALL), highlighting the need for biomarkers and therapeutic targets that limit leukaemic dissemination. We investigated whether histone deacetylase 6 (HDAC6), a regulator of cortactin-dependent actin remodelling, contributes to B-ALL progression and relapse.

**Subjects/Methods:**

HDAC6 expression was analysed in cohorts of 72 paediatric and 54 adult patients with B-ALL and validated in B-ALL cell lines. The functional role of HDAC6 was assessed using pharmacological inhibition and shRNA-mediated knockdown in assays of CXCL12-induced actin remodelling, transendothelial migration, bone marrow colonisation and leukaemic dissemination in vivo. Cortactin-depleted cells were used to determine pathway dependence.

**Results:**

HDAC6 expression was elevated in B-ALL cell lines and patient samples and was significantly associated with relapse in both patient cohorts. Pharmacological inhibition or genetic depletion of HDAC6 impaired CXCL12-induced actin remodelling, transendothelial migration, bone marrow colonisation and leukaemic dissemination. HDAC6 inhibition failed to further reduce migration in cortactin-deficient cells, indicating that HDAC6 promotes leukaemic motility through cortactin.

**Conclusions:**

HDAC6 is a clinically relevant biomarker associated with B-ALL relapse and a critical regulator of cortactin-dependent leukaemic dissemination. Targeting HDAC6 represents a promising therapeutic strategy to limit tissue invasion and reduce relapse.

## Introduction

Acute lymphoblastic leukaemia (ALL) is the most common paediatric malignancy worldwide [1], with B cell ALL (B-ALL) accounting for around 80% of cases [2]. Although most patients achieve complete remission, relapse occurs in ~20% of cases and remains the leading cause of treatment failure [3]. Relapse is typically driven by the outgrowth of therapy-resistant leukaemic clones. The majority of relapses arise in the bone marrow (BM), either in isolation or in combination with central nervous system (CNS), testicular, or other extramedullary involvement [4]. Outcomes after relapse remain poor, largely due to chemotherapy resistance and dissemination of leukaemic cells to peripheral organs [5]. A deeper understanding of the molecular mechanisms underlying leukaemic dissemination and relapse is therefore needed to inform the development of more effective therapies.

The BM microenvironment plays a critical role in regulating leukaemic cell survival, progression and therapeutic response [6, 7]. Leukaemic cells promote a pro-inflammatory niche characterised by reduced CXCL12 production [8, 9], facilitating the egress of B-ALL cells from the BM into the circulation and their dissemination to distant organs, a process guided by the CXCL12/CXCR4 axis [10]. Extramedullary dissemination requires transendothelial migration, which is orchestrated by coordinated signalling through chemokine receptors and adhesion molecules, and dynamic remodelling of the actin cytoskeleton [11]. Actin dynamics are tightly regulated by actin-binding proteins (ABP) including cortactin. We recently demonstrated that cortactin is overexpressed in B-ALL cells and that elevated cortactin levels are associated with enhanced migration, BM colonisation, organ infiltration and relapse [12]. Cortactin activity is regulated by post-translational modification; deacetylation by histone deacetylase 6 (HDAC6) increases its affinity for filamentous actin (F-actin) and promotes efficient cell motility [13].

HDAC6 is a zinc-dependent class II histone deacetylase that localises predominantly to the cytoplasm and regulates cytoskeletal dynamics, in part through deacetylation of α-tubulin, thereby promoting microtubule remodelling required for cell migration [14]. HDAC6 overexpression has been reported in multiple solid tumours, where it correlates with increased invasiveness, metastasis and poor clinical outcome [15–17]. In haematologic malignancies, HDAC6 is upregulated in B-chronic lymphocytic leukaemia (B-CLL), and its pharmacologic inhibition suppresses proliferation and enhances apoptosis [18]. HDAC6 is also overexpressed in primary acute myeloid leukaemia (AML) blasts [19], and promotes leukaemic cell chemotaxis [20].

Here, we hypothesise that HDAC6 promotes B-ALL cell extravasation, organ infiltration and relapse by regulating cytoskeletal dynamics. We demonstrate that HDAC6 is highly expressed in primary paediatric and adult B-ALL blasts and in the REH and RS4:11 cell lines, and that elevated HDAC6 levels are associated with relapse. Functionally, HDAC6 is required for efficient actin polymerisation, transendothelial migration, colonisation of BM spheroids and leukaemic organ invasion in vivo.

## Methods

### Patients and samples

Peripheral blood (PB) and bone marrow (BM) aspirates were obtained from children and adolescents (<18 years) with newly diagnosed B-ALL prior to initiation of therapy or at relapse, in accordance with international and institutional guidelines. Written informed consent was obtained from parents or legal guardians. Paediatric patients were recruited and followed at the Hospital para el Niño del Instituto Materno Infantil del Estado de México (Toluca, protocol CIEICE-03-12-2021); and Hospital para el Niño Poblano (Puebla), Hospital de la Niñez Oaxaqueña (Oaxaca), UMAE–Hospital de Especialidades San José (Puebla), Hospital Infantil de Tlaxcala (Tlaxcala), Hospital Infantil de México ‘Federico Gómez’ (Mexico City) and UMAE-Hospital de Pediatría ‘Dr. Silvestre Frenk Freund’, Centro Médico Nacional Siglo XXI (Mexico City) del Instituto Mexicano del Seguro Social (IMSS, protocols approved by the National Scientific Research and Ethics Committee of IMSS, R-2020-785-177 and R-2020-785-022). The studies were conducted in accordance with the local legislation and institutional requirements. In total, 72 paediatric B-ALL samples were analysed in this study, and the clinical and biological characteristics are summarised in Supplementary Table 3. For a subset of paediatric patients with paired debut–relapse samples (*n* = 16), each time point was analysed as an independent biological sample unless otherwise indicated.

BM aspirates from 54 adults with newly diagnosed B-ALL (Supplementary Table 5) were obtained prior to initiation of therapy and with informed written consent, in accordance with international and institutional guidelines. Adult patients were recruited and followed at Centro Médico Nacional La Raza, IMSS (Mexico City, protocol R-2025-3501-317).

CD19⁺ B cells from healthy donors were purified from PB mononuclear cells obtained from the blood bank of Hospital Juárez (Mexico City, Mexico) and from BM aspirates collected during orthopaedic procedures (non-malignant bone fractures) at Centro Médico Nacional La Raza, IMSS (Mexico City), using the Mojo Sort ™ Human CD19 Selection kit (Biolegend, San Diego, CA) according to the manufacturer’s instructions.

### Cell culture and treatments

The B-ALL cell lines REH (ATCC, CRL-8286) and RS4:11 (ATCC, CRL-1873), and the stromal cell line HS5 (ATCC CRL-11882) were cultured in RPMI-1640 or DMEM (Sigma/Merck), respectively, supplemented with 10% foetal bovine serum (FBS). HDAC6-depleted REH cells were generated by lentiviral transduction using the HDAC6 Human shRNA Plasmid Kit (OriGene, Cat. #TL312491).

Human umbilical vein endothelial cells (HUVEC) were isolated from discarded umbilical cords as described [21] and cultured in ECM (ScienCell, Carlsbad, CA) supplemented with 10% FBS.

Primary CD19^+^ B cells from healthy donors and B-ALL patients were cultured in RPMI-1640 supplemented with 10% FBS. Progenitor cells (CD34⁺CD19⁻), pro-B cells (CD34⁺CD19⁺) and pre-B cells (CD34⁻CD19⁺) were identified by flow cytometry as described in the supplement.

For pharmacologic inhibition of HDAC6, primary BM-derived B-ALL blasts and REH cells were treated with tubastatin A (TubA; 10 or 20 μM; Merck) for the indicated times. Control cells received equivalent volumes of DMSO.

### Transendothelial migration assay

HUVEC were grown to confluence on gelatin-treated 5 µm-pore transwell filters (Corning). REH cells or patient-derived BM B-ALL cells (1.5 × 10⁴) were pretreated with TubA or DMSO, resuspended in RPMI-1640, and added to the upper chamber. CXCL12 (100 ng/mL; PeproTech) was placed in the lower chamber as chemoattractant. After 4 h at 37 °C, transmigrated cells were collected from the lower chamber and counted using a Neubauer hemocytometer with trypan blue exclusion.

### In vitro BM spheroid colonisation assay

HS5 cells were seeded in 1% agarose-coated plates to generate spheroids [9]. REH or patient-derived B-ALL cells were labelled with CFDA (Invitrogen) and co-cultured with spheroids overnight. Spheroids were recovered, washed with PBS-EDTA (5 mM), dissociated using TrypLE and mechanical dissociation and analysed by flow cytometry. Cells recovered from within spheroids (IN) and from the supernatant (OUT) were analysed for HDAC6 expression.

For competition assays, DMSO-treated cells were labelled with CFDA and TubA–treated cells with CellTrace Violet (CTV). The percentage of cells colonising spheroids was quantified by flow cytometry. For imaging, spheroids were fixed, mounted in DABCO and imaged by confocal microscopy. Cell numbers within spheroids were quantified using IMARIS software (Oxford Instruments, Abingdon, UK).

### Leukaemia xenograft model

REH cells (3 × 10⁶) were injected intravenously into non-irradiated male NSG mice (Jackson Laboratories). Mice received TubA (50 mg/kg/day, intraperitoneally) every other day for 27 days, starting on the day of leukaemic cell injection to specifically evaluate early homing and initial organ colonisation. Control mice received vehicle (DMSO). Body weight was monitored three times per week. Upon clinical disease manifestation in control mice, animals were euthanized and BM, PB, liver, spleen, brain, lung and testes were harvested and mechanically disaggregated. Single-cell suspensions were analysed for human CD45⁺ leukaemic cells by flow cytometry as described above. All animal experiments were approved by the CINVESTAV IACUC (Protocol 0263–18) and conducted in accordance with Mexican regulatory guidelines.

Additional methods are described in the supplement.

## Results

### HDAC6 is overexpressed in patient-derived B-ALL cells and correlates with relapse

**Fig. 1:**
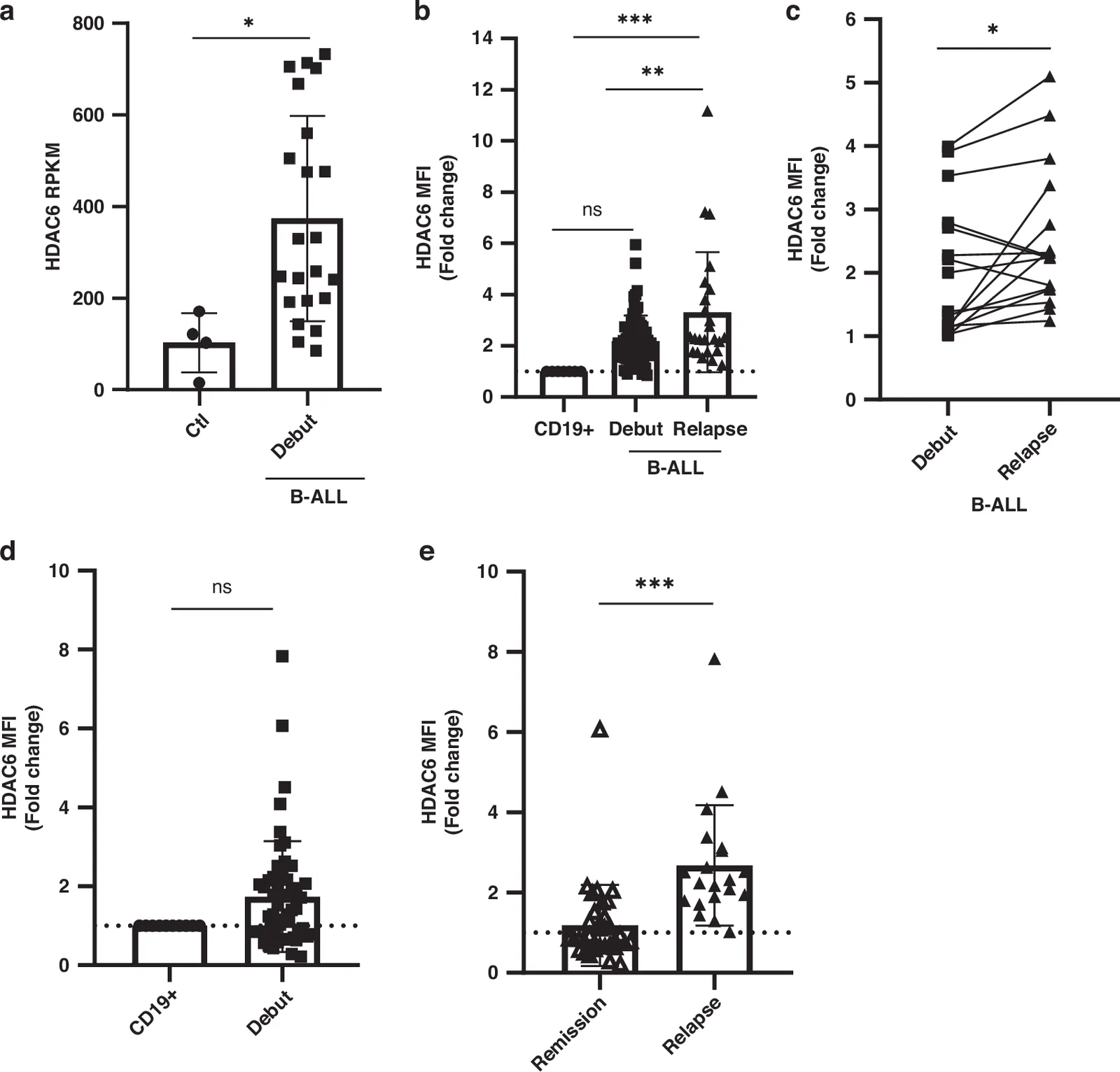
HDAC6 mRNA and protein levels are elevated in primary leukaemic B cells compared with normal B cells. **a** HDAC6 expression (RPKM, reads per kilobase per million) determined by RNA-seq in paediatric B-ALL patients (*n* = 22) and controls (*n* = 4). Each dot represents one individual. Unpaired two-tailed Student’s *t* test. **b** Flow cytometric analysis of HDAC6 protein levels in paediatric B-ALL at diagnosis (debut; *n* = 64) and relapse (*n* = 24), normalised to healthy CD19⁺ B cells (set to 1, dotted line). One-way ANOVA. **c** HDAC6 protein levels in paired debut–relapse samples from paediatric B-ALL patients (*n* = 16), normalised to healthy CD19⁺ B cells (set to 1). Paired two-tailed Student’s *t* test. **d** HDAC6 protein levels in adult B-ALL at debut (*n* = 54), normalised to healthy CD19⁺ B cells (set to 1, dotted line). Unpaired two-tailed Student’s *t* test. **e** HDAC6 protein levels in adult B-ALL at debut stratified by clinical outcome (remission versus relapse), normalized to healthy CD19⁺ B cells (set to 1, dotted line). Mann–Whitney *U* test. Data in (**b**–**e**) are shown as fold change of mean fluorescence intensity (MFI); error bars indicate standard deviation. *p < 0.05; **p < 0.01; ***p < 0.001; ns not significant.

Because our primary objective was to define the clinical relevance of HDAC6 in paediatric B-ALL, we first analysed HDAC6 expression in patient cohorts. Analysis of RNA-seq data from 22 paediatric B-ALL patients (Supplementary Table 1) showed significantly higher HDAC6 expression compared with healthy controls (Fig. 1a). However, non-parametric Mann–Whitney *U* tests revealed no significant associations between HDAC6 expression and sex, risk group, hepatomegaly, splenomegaly, adenomegaly, or death (Supplementary Table 2). Pearson’s correlation analysis further showed that HDAC6 expression did not correlate with bone marrow (BM) blast percentage, leucocyte count, haemoglobin concentration, or platelet count (Supplementary Fig. 1).

We next assessed HDAC6 protein expression by flow cytometry in bone marrow samples from an independent cohort of 72 paediatric B-ALL patients (Supplementary Table 3). In this cohort, HDAC6 protein levels tended to be higher in B-ALL blasts from newly diagnosed patients (debut) than in normal CD19⁺ B cells (Fig. 1b), reflecting the heterogeneity of HDAC6 expression at diagnosis. Notably, HDAC6 expression was significantly increased in samples from relapsed patients compared with both normal CD19⁺ B cells and cells at debut (Fig. 1b). Statistical analysis of the clinical data showed a significant association between high HDAC6 protein levels and relapse (Supplementary Table 4). In contrast, no associations were observed with sex, risk group, immunophenotype, steroid response, infections, neutropenia, thrombocytopenia, anaemia, hepatomegaly, adenomegaly, splenomegaly, or death (Supplementary Table 4). HDAC6 protein levels negatively correlated with bone marrow blast percentage but showed no correlation with blast percentage in peripheral blood (PB), lactate dehydrogenase (LDH), leucocyte counts, haemoglobin concentration, or platelet counts (Supplementary Fig. 2). Importantly, HDAC6 protein levels were significantly higher at relapse in 16 paired debut-relapse samples from paediatric B-ALL patients (Fig. 1c), indicating a more prominent role for HDAC6 during relapse.

Analysis of a cohort of 54 adult B-ALL patients (Supplementary Table 5) also showed a trend to higher HDAC6 levels at debut compared with healthy B cells, mirroring the trend observed in paediatric B-ALL (Fig. 1d), further demonstrating the biological heterogeneity at debut in both paediatric and adult patients. Importantly, adult patients who subsequently relapsed exhibited significantly higher HDAC6 levels at debut than those who remained in remission (Fig. 1e), supporting the potential of HDAC6 as biomarker for early determination of relapse risk in adult B-ALL. Together, these findings demonstrate that HDAC6 overexpression is strongly associated with relapse in both paediatric and adult B-ALL.

### The B-ALL cell line REH exhibits elevated HDAC6 levels compared with normal B cells

**Fig. 2:**
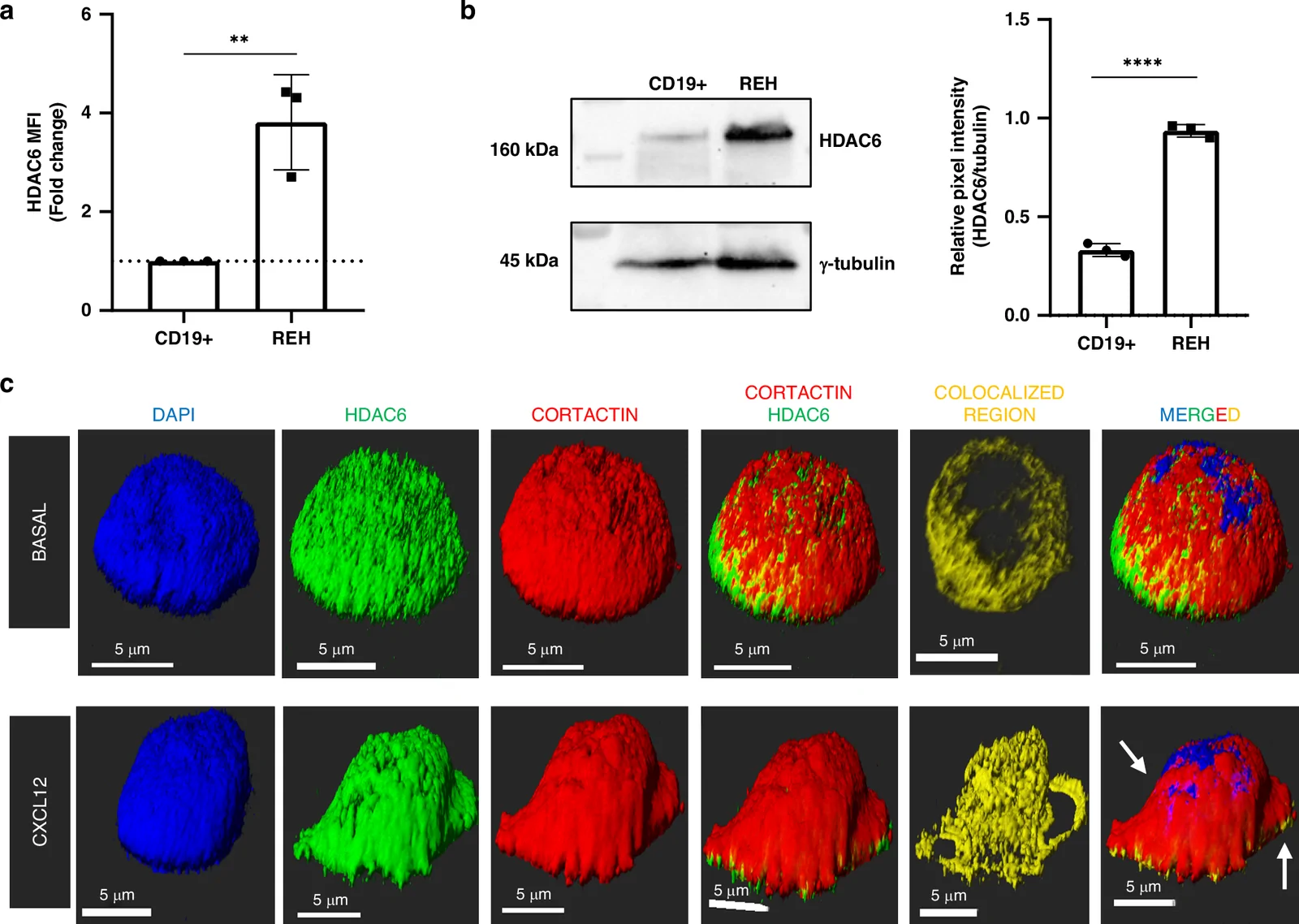
HDAC6 is overexpressed in REH cells and localises predominantly to the cytoplasm. **a** Flow cytometric quantification of HDAC6 protein levels in REH cells normalized to healthy bone marrow (BM) CD19⁺ B cells (set to 1, dotted line). Unpaired two-tailed Student’s *t* test; *n* = 3. **b** Representative immunoblot and densitometric quantification of HDAC6 in CD19⁺ B cells and REH cells, showing the expected 160-kDa band. γ-Tubulin served as loading control. Unpaired two-tailed Student’s *t* test; *n* = 3. Error bars in (**a**, **b**) indicate standard deviation. **p < 0.01; ****p ≤ 0.0001. **c** 3D surface rendering of confocal z-stacks was performed using Imaris to visualise the spatial distribution of HDAC6 (green) and cortactin (red) in REH cells under basal and CXCL12 stimulation (100 ng/mL, 20 min). A masked colocalization channel (yellow) was generated from automatically thresholded surfaces to identify overlapping voxels. Representative images are shown; at least 20 REH cells were examined across three independent experiments. Under basal conditions, HDAC6 and cortactin exhibited partial spatial overlap. CXCL12 stimulation induced polarised morphology with lamellipodia-like protrusions and increased HDAC6–cortactin colocalization at the leading edge. Arrows indicate the leading and trailing edges.

To investigate the functional consequences of HDAC6 overexpression observed in patients, we used the B-ALL cell line REH. First, we analysed HDAC6 protein levels by flow cytometry in REH cells compared with healthy BM-derived CD19^+^ B cells and found that leukaemic cells contain significantly higher HDAC6 levels (Fig. 2a). Western blot analysis confirmed this finding and showed the expected molecular weight of HDAC6 at 160 kDa (Fig. 2b). Importantly, CD19^+^ B cells from healthy BM and CD19^+^ B cells from healthy PB express similar HDAC6 levels (Supplementary Fig. 3A), indicating that the observed differences were specific to leukaemic cells rather than tissue origin. This finding also validates the use of healthy PB-derived CD19^+^ B cells as controls, given the very limited availability of healthy BM samples.

Consistent HDAC6 overexpression was also observed in RS4:11, another B-ALL cell line, with no significant differences in HDAC6 levels between REH and RS4:11 cells (Supplementary Fig. 3B, C).

3D surface rendering of confocal z-stacks revealed that HDAC6 localised predominantly to the cytoplasm of REH cells (Fig. 2c), in agreement with previous reports [22]. Under basal conditions, cortactin showed partial colocalization with HDAC6, as visualised in the masked colocalization channel (Fig. 2c, upper panel). Upon CXCL12 stimulation, REH cells displayed polarised morphology with lamellipodia-like protrusions, with enrichment of both HDAC6 and cortactin at the leading edge (Fig. 2c, lower panel). These findings suggest that HDAC6 may contribute to cortactin-dependent cytoskeletal remodelling during leukaemic cell migration.

### HDAC6 inhibition reduces leukaemic B-cell migration through F-actin polymerisation and CXCR4/VLA-4 expression

To assess the functional impact of HDAC6 inhibition in REH cells, we first established appropriate conditions for treatment with TubA, a selective HDAC6 inhibitor [23, 24]. We found that the IC_50_ value of TubA was 94 µM after 24 h (Supplementary Fig. 4A), and that treatment with 20 µM of TubA for 3 h did not induce apoptosis (Supplementary Fig. 4B). Inhibition of HDAC6 with 20 µM of TubA in REH cells increased acetylation levels of both cortactin and α-tubulin without changing HDAC6 levels (Supplementary Fig. 4C). Inhibitory effects were still visible after 24 h (Supplementary Fig. 4D). Cortactin and α-tubulin are important proteins regulating cell migration [25, 26] and have been reported as HDAC6 substrates in different cell lines [14, 27]. Our findings demonstrate that cortactin and tubulin are also HDAC6 targets in REH cells.

**Fig. 3:**
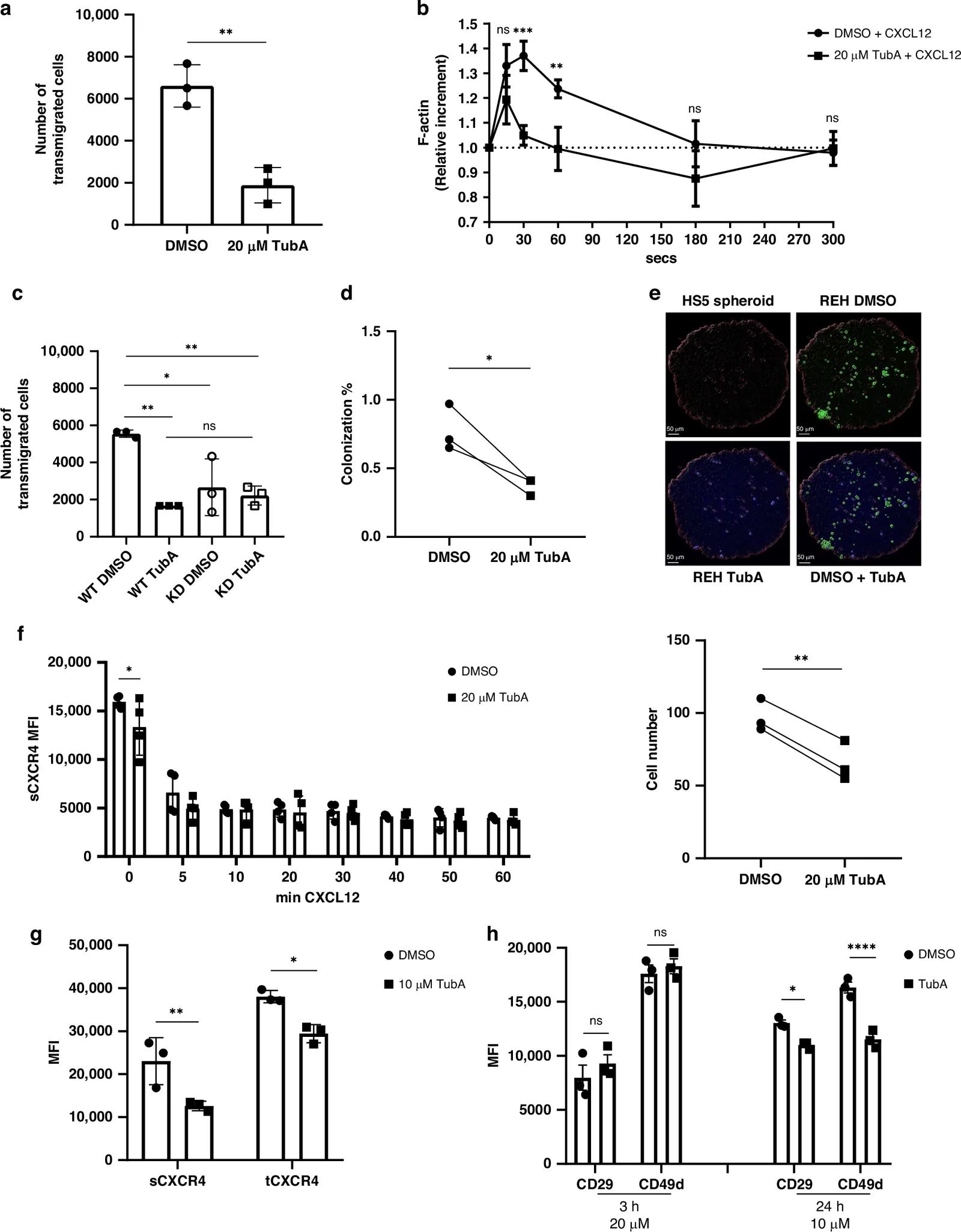
Pharmacologic inhibition of HDAC6 impairs leukaemic B-cell migration. **a** Transendothelial migration of REH cells across HUVEC monolayers toward CXCL12 (100 ng/mL, 4 h) following treatment of only REH cells with TubA (TubA; 20 µM, 1 h) or DMSO. Unpaired two-tailed Student’s *t* test; *n* = 3. **b** Dynamics of F-actin polymerisation in REH cells pretreated with TubA or DMSO and stimulated with CXCL12 for the indicated times. Cells were immediately fixed and stained with phalloidin. Data are shown relative to time 0 (set to 1; dotted line). Two-way ANOVA; *n* = 3. **c** Transendothelial migration of wild-type (WT) and cortactin-depleted REH cells treated with TubA (20 µM, 3 h) or DMSO toward CXCL12 (100 ng/mL, 4 h). Migrated cells were counted using a Neubauer chamber. Two-way ANOVA; *n* = 4. **d** Colonisation of HS5-MSC spheroids by REH cells after 24 h co-culture. Control cells (DMSO) were CFDA-labelled and TubA-treated cells were CTV-labelled. Colonisation was quantified by flow cytometry. Paired two-tailed Student’s *t* test; *n* = 3. **e** Confocal imaging and quantification of REH cells (CFDA-labelled DMSO cells in green; CTV-labelled TubA-treated cells in blue) within HS5-MSC spheroids. Representative images are shown. Scale bar = 50 µm. Paired two-tailed Student’s *t* test; *n* = 3. **f** Surface CXCR4 expression in non-permeabilized REH cells after TubA treatment (20 µM, 3 h) and CXCL12 stimulation. Two-way ANOVA; *n* = 4. **g** Surface and total CXCR4 expression after 24 h treatment with TubA (10 µM). Two-way ANOVA; *n* = 3. **h** Surface CD29 and CD49d (VLA-4) expression after 3 h (20 µM) and 24 h (10 µM) of TubA treatment. Two-way ANOVA; *n* = 3. Data are shown as MFI ± standard deviation. *p < 0.05; **p < 0.01; ***p < 0.001; ****p ≤ 0.0001; ns not significant.

We next analysed the role of HDAC6 in REH cell migration. HDAC6 inhibition significantly reduced CXCL12-induced transmigration of REH cells across human umbilical vein endothelial cell (HUVEC) monolayers compared with DMSO-treated controls (Fig. 3a), indicating that HDAC6 activity is required for efficient transendothelial migration (TEM). Because TEM critically depends on F-actin dynamics, we assessed actin polymerisation following CXCL12 stimulation. Whereas control cells rapidly polymerised actin, TubA–treated REH cells exhibited significantly reduced F-actin formation (Fig. 3b).

Cortactin stabilises actin filaments [28], regulates TEM [29] and is a main target of HDAC6 [27]. To determine whether HDAC6 promotes TEM through cortactin, we inhibited HDAC6 in stable cortactin-knockdown (KD) REH cells. As previously reported [12], vehicle-treated cortactin-KD cells displayed significantly reduced transmigration compared with wild-type (WT) REH cells (Fig. 3c). Notably, HDAC6 inhibition in cortactin-KD cells did not further reduce TEM compared with DMSO-treated cortactin-KD cells or TubA-treated WT cells (Fig. 3c). These findings indicate that HDAC6 promotes TEM predominantly via cortactin deacetylation, which enhances cortactin binding to F-actin [27] and stabilises newly polymerised actin filaments in response to CXCL12.

The BM microenvironment provides essential cues for leukaemic cell survival and dissemination. CXCL12, constitutively produced by BM mesenchymal stromal cells (MSCs), regulates homing and retention of circulating normal and leukaemic cells [7]. Three-dimensional MSC spheroids recapitulate key features of the leukaemic niche, including cell–cell interactions, extracellular matrix deposition and hypoxia [30–32]. To determine whether HDAC6 contributes to leukaemic cell homing to the BM niche, we performed competition assays using MSC spheroids formed by the HS5 cell line. Flow cytometric analysis revealed that TubA-treated REH cells colonised MSC spheroids significantly less efficiently than DMSO-treated controls (Fig. 3d). Confocal microscopy and Imaris-based quantification confirmed a significant reduction in the number of HDAC6-inhibited REH cells within BM spheroids (Fig. 3e), demonstrating that HDAC6 activity is required for efficient homing of B-ALL cells to the BM microenvironment.

Transendothelial migration and bone marrow homing of leukaemic B cells depend on coordinated signalling through chemokine receptors and integrins. We observed significantly reduced surface expression of CXCR4 in TubA-treated REH cells compared with DMSO-treated controls (Fig. 3f). In contrast, ligand-induced internalisation of CXCR4 in response to CXCL12 occurred at comparable rates in both treatment groups (Fig. 3f), indicating that HDAC6 inhibition does not impair receptor endocytosis.

After 24 h of treatment with 10 µM TubA, REH cell viability remained high (Supplementary Fig. 5A), and both surface and total CXCR4 protein levels were significantly decreased (Fig. 3g), suggesting that HDAC6 activity contributes to maintenance of CXCR4 expression. Prolonged HDAC6 inhibition with 10 µM TubA also significantly reduced surface expression of CD49d and CD29 (Fig. 3h), the α4 and β1 subunits of the integrin very late antigen-4 (VLA-4), which is critical for B-cell adhesion and BM retention. This reduction was not detectable after short-term (3 h) treatment with 20 µM TubA (Fig. 3h), indicating a time-dependent effect consistent with altered receptor trafficking and/or protein stability rather than acute internalisation.

Collectively, these data show that HDAC6 drives leukaemic B cell migration by stabilising F-actin and regulating CXCR4 and VLA-4 expression to support the migratory and homing capacity of B-ALL cells.

### Primary B-ALL cells require HDAC6 activity for migration and bone marrow colonisation

**Fig. 4:**
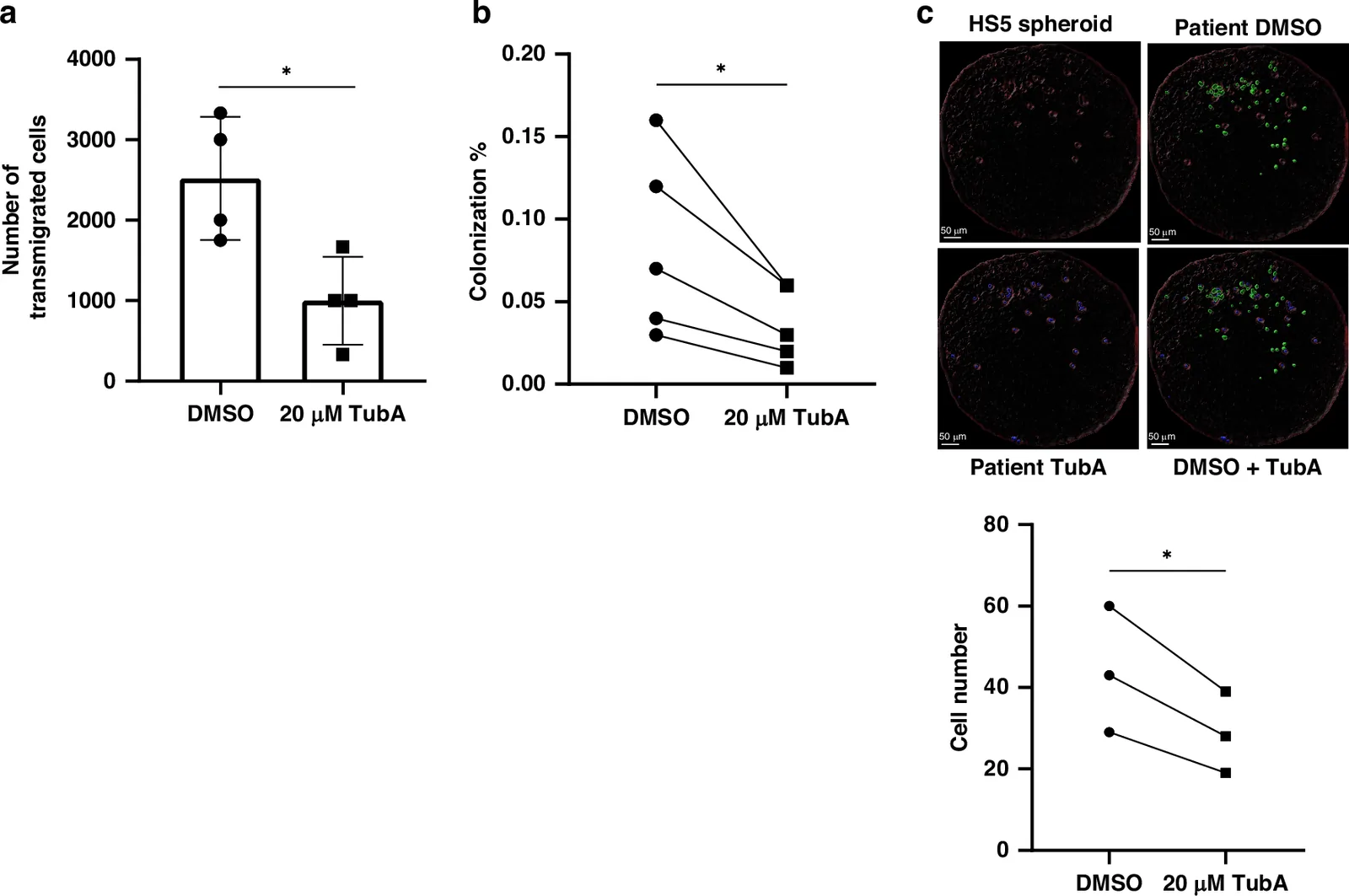
HDAC6 inhibition reduces the migratory capacity of primary B-ALL cells. **a** Transendothelial migration of paediatric patient–derived B-ALL cells across HUVEC monolayers toward CXCL12 (100 ng/mL, 4 h) after TubA treatment (20 µM, 3 h) or DMSO. Unpaired two-tailed Student’s *t* test; *n* = 4. **b** Colonisation of HS5-MSC spheroids by paediatric B-ALL cells after 24 h co-culture. Control cells were labelled with CFDA and TubA-treated cells with CTV. Paired two-tailed Student’s *t* test; *n* = 5. **c** Confocal imaging and quantification of paediatric B-ALL cells (CFDA-labelled DMSO cells in green; CTV-labelled TubA-treated cells in blue) within HS5-MSC spheroids. Representative images are shown. Scale bar = 50 µm. Paired two-tailed Student’s *t* test; *n* = 3. Error bars indicate standard deviation. *p < 0.05.

We next validated these findings obtained in REH cells using patient-derived B-ALL cells. HDAC6 inhibition significantly reduced CXCL12-induced transmigration of primary B-ALL cells across HUVEC monolayers (Fig. 4a). Consistently, TubA-treated primary B-ALL cells exhibited impaired colonisation of BM-MSC spheroids compared with vehicle-treated cells, as determined by flow cytometry (Fig. 4b) and confocal microscopy (Fig. 4c). Together, these results support that HDAC6 activity is required for efficient migration and BM homing of patient-derived leukaemic B cells.

### The bone marrow microenvironment enhances HDAC6 expression

**Fig. 5:**
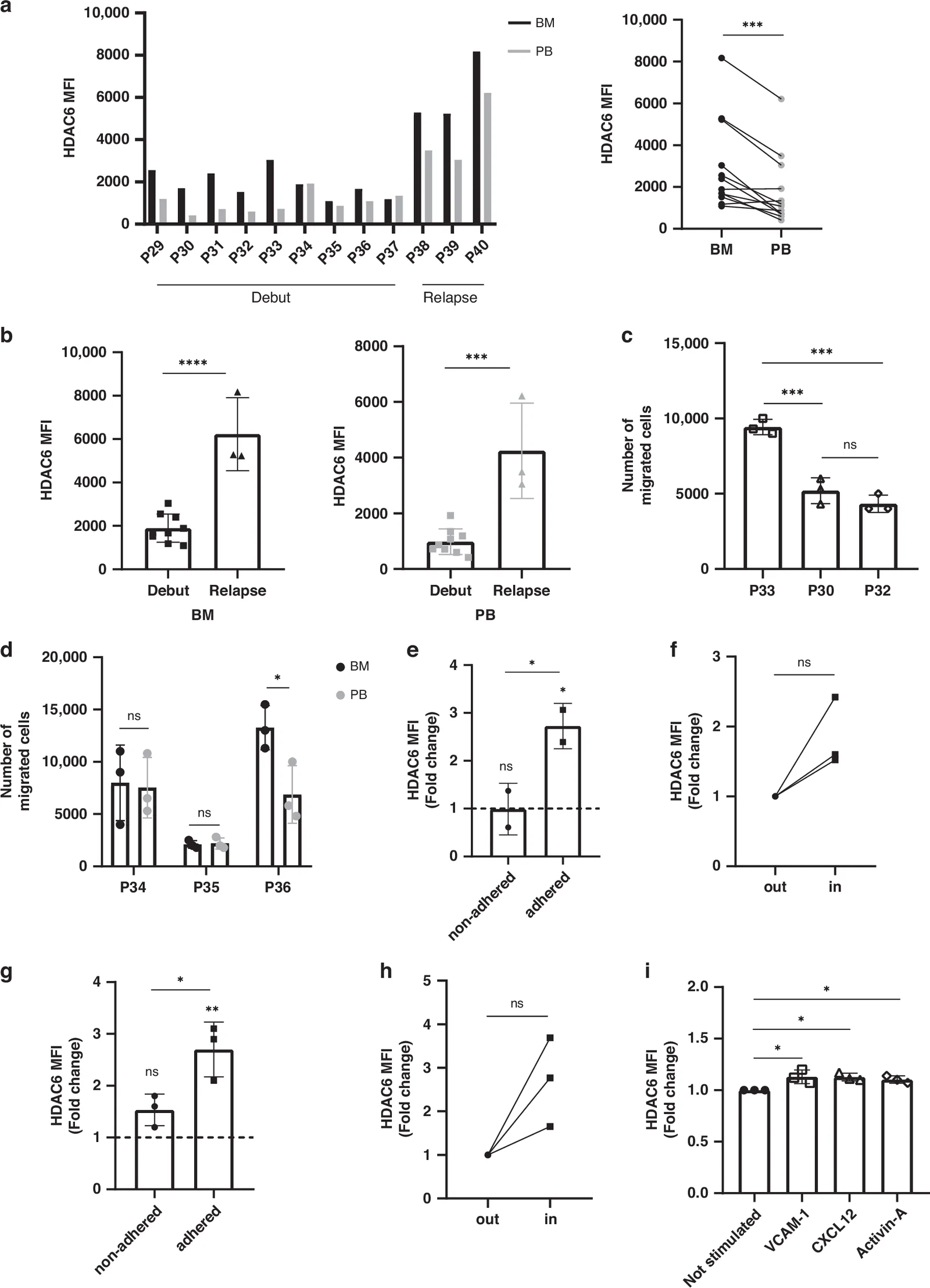
The leukaemic bone marrow microenvironment induces HDAC6 upregulation. **a** HDAC6 protein levels in paired bone marrow (BM) and peripheral blood (PB) blasts from newly diagnosed (debut, *n* = 9) and relapsed (*n* = 3) paediatric B-ALL patients. Each patient contributed matched BM and PB samples (*n* = 12 paired samples). Paired two-tailed Student’s *t* test. **b** Comparison of HDAC6 levels in BM- and PB-derived blasts from newly diagnosed (debut) and relapsed patients. Unpaired two-tailed Student’s *t* test. **c** Chemotaxis of patient-derived BM blasts toward CXCL12 (100 ng/mL, 4 h). One-way ANOVA. **d** Chemotaxis of paired BM and PB blasts toward CXCL12. One-way ANOVA. **e** HDAC6 levels in adhered and non-adhered patient-derived blasts following co-culture with HS5-MSC monolayers for 24 h, shown as fold change relative to cells cultured alone (set to 1, dotted line,). One-way ANOVA; *n* = 2 independent experiments. **f** HDAC6 levels in patient-derived blasts colonising HS5-MSC spheroids (‘in’) versus non-colonising cells (‘out’). Paired two-tailed Student’s *t* test; *n* = 3. HDAC6 levels in REH cells following co-culture with HS5-MSC monolayers (**g**) or HS5-MSC spheroids (**h**). One-way ANOVA (**g**); paired two-tailed Student’s *t* test (**h**); *n* = 3. **i** HDAC6 expression in REH cells cultured on VCAM-1–Fc (500 ng/mL) or stimulated with CXCL12 (100 ng/mL) or Activin-A (50 ng/mL) for 24 h. One-way ANOVA; *n* = 3. Error bars indicate standard deviation. *p < 0.05; **p < 0.01; ***p < 0.001; ****p ≤ 0.0001; ns not significant.

Because leukaemic blasts in the bone marrow are exposed to niche-derived cues that regulate migration and retention, we next analysed HDAC6 levels in paired BM-PB paediatric samples to determine whether HDAC6 upregulation is preferentially induced within the BM microenvironment. In 9 of the 12 patients analysed from the Hospital Infantil de Mexico ‘Federico Gómez’ (Mexico City) (Supplementary Table 1), HDAC6 levels were higher in BM-derived blasts than in matched PB-derived blasts (Fig. 5a), suggesting that HDAC6 upregulation occurs within the BM microenvironment and may contribute to leukaemic cell egress into the circulation. Consistently, blasts from relapsed patients exhibited higher HDAC6 levels in both BM and PB compared with those from patients at debut (Fig. 5b), further supporting a link between high HDAC6 expression and disease aggressiveness.

To determine whether HDAC6 expression correlates with migratory behaviour, we next examined CXCL12-induced migration in patient samples with different HDAC6 levels. BM-derived blasts from a patient at debut with the highest HDAC6 levels (P33) exhibited greater CXCL12-induced migration than blasts from patients with lower HDAC6 expression (P30, P32) (Fig. 5c). Analysis of paired BM and PB samples from three additional patients further confirmed that higher migratory capacity correlated with higher HDAC6 levels in BM blasts (e.g. P36 BM; Fig. 5d), consistent with a functional role for HDAC6 in promoting leukaemic cell migration.

To directly assess whether the BM niche promotes HDAC6 expression, primary B-ALL cells were co-cultured for 24 h with the BM-MSC HS5. Adhesion to HS5 MSCs resulted in a significant increase in HDAC6 levels, whereas non-adherent cells did not exhibit such changes (Fig. 5e), indicating that direct contact is a dominant driver of HDAC6 upregulation in patient-derived blasts. Consistently, patient-derived B-ALL cells that successfully colonised BM-MSC spheroids tended to express higher HDAC6 levels than non-colonising cells (Fig. 5f). Due to limited availability of patient-derived blasts, these experiments could not be repeated extensively to reach statistical significance.

REH cells showed a similar pattern. REH cells adherent to HS5 exhibited significantly increased HDAC6 levels compared with REH cells cultured alone and non-adherent REH cells recovered from the supernatant of the co-culture (Fig. 5g), supporting that direct cell–cell contact with MSCs is a dominant driver of HDAC6 upregulation. REH cells that successfully colonized BM-MSC spheroids expressed higher HDAC6 levels than non-colonising cells (Fig. 5h).

Because MSCs express CXCL12 and VCAM-1, key mediators of leukaemic cell adhesion, retention and mobilisation within the BM niche, we evaluated whether these factors contribute to HDAC6 induction. CXCL12 stimulation and adhesion to VCAM-1 each modestly increased HDAC6 levels in REH cells (Fig. 5i). Activin-A, a TGF-β family member produced by MSCs and recently implicated in promoting B-ALL migration [33], also induced a modest increase in HDAC6 expression (Fig. 5i). However, none of these individual stimuli recapitulated the robust HDAC6 upregulation observed in 2D and 3D MSC co-culture systems, indicating that MSC-mediated induction of HDAC6 likely requires a complex combination of signals.

### HDAC6 depletion in B-ALL cells impairs leukaemic cell migration and BM colonisation

**Fig. 6:**
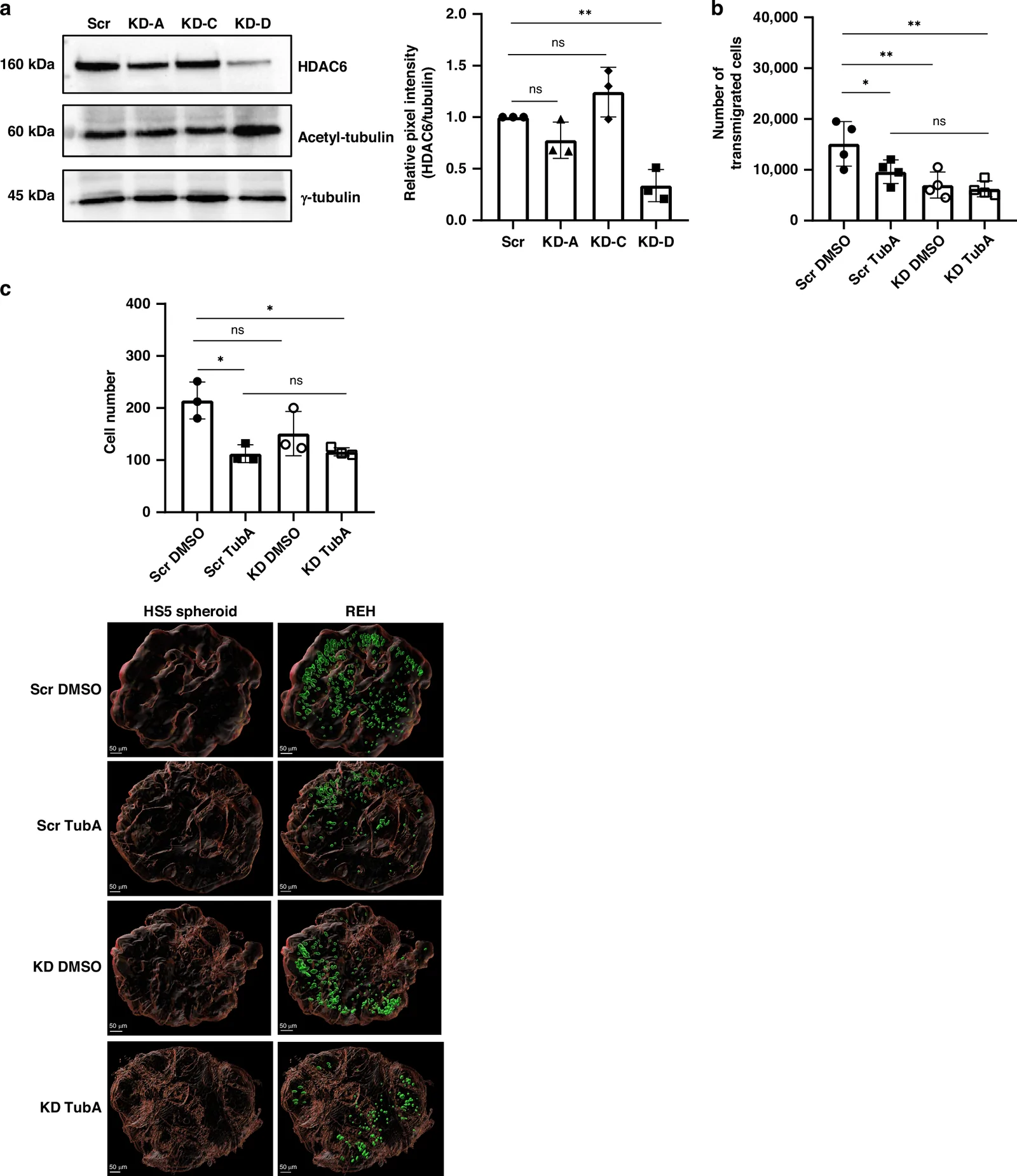
Genetic depletion of HDAC6 impairs leukaemic B-cell migration. **a** Representative immunoblot and densitometric quantification of HDAC6 in REH cells transduced with three independent HDAC6-targeting shRNAs (A, C, D) or scramble control (Scr). Acetylated tubulin and γ-tubulin served as functional and loading controls, respectively; *n* = 3. **b** Transendothelial migration of Scr and HDAC6-depleted REH cells treated with TubA (20 µM, 3 h) or DMSO toward CXCL12 (100 ng/mL, 4 h). Two-way ANOVA; *n* = 4. **c** Confocal imaging and quantification of HDAC6-depleted REH cells colonising HS5-MSC spheroids after TubA or DMSO treatment. Representative images are shown. Scale bar = 50 µm. Two-way ANOVA; *n* = 3. Error bars indicate standard deviation. *p < 0.05; **p < 0.01; ns not significant.

To further validate the role of HDAC6 in leukaemic cell migration, we generated HDAC6-depleted REH cells using shRNA (Fig. 6a). Among the constructs tested, HDAC6 KD-D achieved the most robust knockdown (~70%) and induced an increase in acetyl-tubulin levels, confirming functional inhibition of HDAC6 activity. Therefore, these cells were used for all subsequent functional experiments. Genetic depletion of HDAC6 significantly reduced CXCL12-induced transmigration of REH cells across HUVEC monolayers compared with scrambled control cells (Fig. 6b). Importantly, pharmacological inhibition of HDAC6 with TubA did not further decrease transmigration in HDAC6-depleted cells relative to DMSO-treated HDAC6-knockdown cells or TubA–treated scramble controls, indicating that HDAC6 activity is required for efficient transendothelial migration in REH cells and that the TubA-mediated effect occurs through HDAC6 (Fig. 6b). Consistent with these findings, HDAC6-depleted REH cells also exhibited significantly impaired colonisation of BM MSC spheroids compared with control cells (Fig. 6c). Together, these data show that TubA phenocopies the effects of genetic HDAC6 depletion, supporting target specificity and a critical role for HDAC6 in leukaemic cell migration and homing to the BM.

### TubA treatment in xenotransplanted mice limits organ invasion by leukaemic B cells

**Fig. 7:**
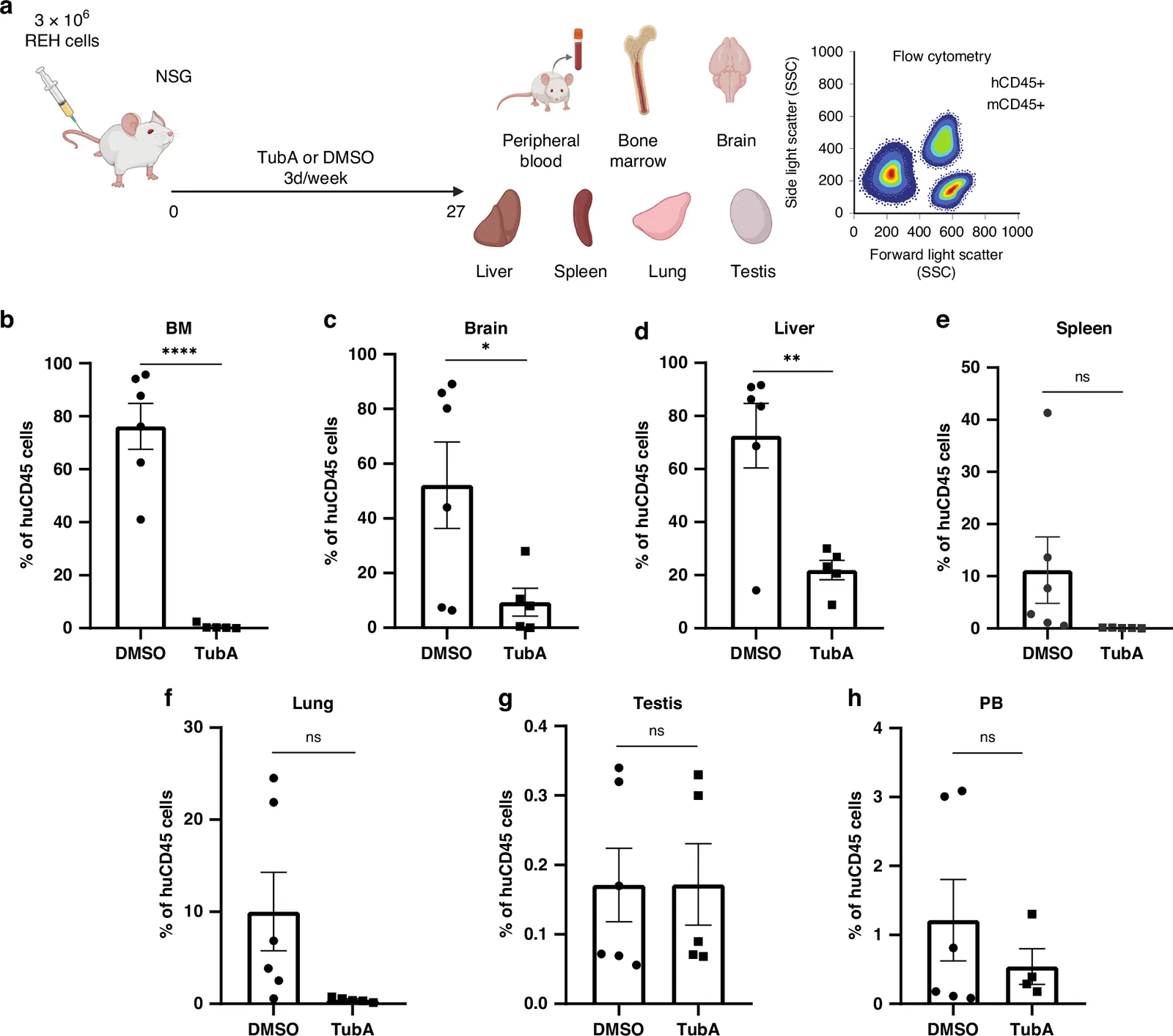
TubA treatment reduces leukaemic cell infiltration in vivo. **a** Representative schematic of the in vivo xenograft experiment. NSG mice were injected intravenously with 3 × 10⁶ REH cells and treated with TubA (50 mg/kg; *n* = 5) or DMSO (*n* = 6) every other day for 27 days. At the end of treatment, peripheral blood, bone marrow, brain, liver, spleen, lung and testis were collected for analysis. Human leukaemic burden was quantified by flow cytometry using hCD45⁺ and mCD45⁺ gating strategies based on forward (FSC) and side (SSC) scatter parameters. Quantification of human leukaemic infiltration (hCD45⁺ cells) in bone marrow (**b**), brain (**c**), liver (**d**), spleen (**e**), lung (**f**), testis (**g**) and peripheral blood (**h**). Data represent the percentage of infiltrating hCD45⁺ cells among live cells. Error bars indicate SEM. Unpaired two-tailed Student’s *t* test. *p < 0.05; **p < 0.01; ****p ≤ 0.0001; ns not significant.

To assess the in vivo relevance of pharmacological HDAC6 inhibition, we employed a xenograft model of B-ALL by transplanting human REH cells into NSG mice and treating animals with either TubA or vehicle (Fig. 7a). TubA administration was initiated at the time of leukaemic cell injection to assess early homing and initial organ colonisation, processes that occur within the first hours to days after intravenous delivery. During the first 3 weeks of the experimental period, overt signs of disease or weight loss were not observed by the veterinarians. Disease was established in all vehicle-treated mice by day 27, evidenced by reduced activity and hind-limb paralysis as observed by veterinarians. In contrast, none of the TubA-treated mice exhibited overt signs of disease at this time point.

TubA treatment markedly impaired homing of REH cells to the bone marrow (BM) (Fig. 7b) and significantly reduced leukaemic infiltration of the brain (Fig. 7c) and liver (Fig. 7d). A trend toward reduced leukaemic burden was also observed in the spleen (Fig. 7e) and lungs (Fig. 7f). In contrast, no differences were detected in testicular infiltration (Fig. 7g), suggesting that leukaemic colonisation of this sanctuary site may occur independently of HDAC6 activity. Circulating REH cells in peripheral blood were modestly reduced in TubA-treated mice (Fig. 7h). Representative flow cytometry gating of murine (mCD45⁺) and human (hCD45⁺) leukaemic populations is shown in Supplementary Fig. 6A.

In a separate experiment, TubA administration was initiated after leukaemic cells were already established in the BM, a consistent trend toward reduced leukaemic infiltration across multiple organs was observed compared with vehicle-treated controls (Supplementary Fig. 6B). Moreover, TubA treatment was associated with a trend toward improved survival (Supplementary Fig. 6C), although inter-animal variability limited statistical power and precluded definitive conclusions.

Collectively, these in vivo data corroborate a critical role for HDAC6 in promoting the migratory and invasive behaviour of B-ALL cells and support HDAC6 as a potential therapeutic target to limit organ infiltration in B-ALL.

## Discussion

HDAC6 is a cytoplasmic deacetylase [34, 35] that regulates cell migration and is overexpressed in many solid tumours, where it correlates with metastatic potential [22, 36]. In haematologic malignancies, HDAC6 upregulation has been reported in B-CLL and AML [18–20]; however, its functional role in leukaemia remained unclear. Here, we demonstrate that HDAC6 is significantly upregulated in paediatric and adult B-ALL. Although HDAC6 levels at diagnosis were heterogeneous, the highest levels were consistently detected in relapsed samples. This suggests that HDAC6^high^ leukaemic blasts may possess a selective advantage that facilitates persistence in protective BM niches, chemoresistance and eventual relapse. Supporting this, an independent adult cohort revealed that patients who subsequently relapsed exhibited significantly higher HDAC6 levels at debut than those who remained in remission. These findings position HDAC6 as a promising biomarker of relapse risk.

We further demonstrate that HDAC6 is highly expressed in the cytoplasm of REH cells, where it co-localises with cortactin and regulates leukaemic cell migration and organ infiltration. Both HDAC6 depletion and pharmacological inhibition reduced TEM, MSC-spheroid colonisation and in vivo organ infiltration. Mechanistically, these effects were associated with impaired CXCL12-induced, cortactin-dependent F-actin polymerisation. Cortactin deacetylation by HDAC6 enhances its affinity for F-actin and stabilises branched actin filaments, whereas hyperacetylated cortactin exhibits reduced actin binding and impaired motility [27, 28, 37]. Thus, HDAC6-mediated cortactin deacetylation provides a plausible mechanism by which HDAC6 promotes leukaemic cell migration [38].

HDAC6 may also influence leukaemic trafficking through regulation of CXCR4. CXCR4 stability depends on the heat shock protein 90 (HSP90) [39], and HDAC6 deacetylates HSP90 [40], thereby maintaining its chaperone activity. Consistent with this, we found that selective HDAC6 inhibition reduced both surface and total CXCR4 levels in B-ALL cells, suggesting an additional mechanism by which HDAC6 inhibition attenuates CXCL12-directed migration.

Beyond CXCR4, HDAC6 may regulate membrane availability of CXCR4 and VLA-4 through its role in endosomal transport. HDAC6 associates with endosomal compartments and modulates receptor trafficking via tubulin deacetylation [41]. Pan-HDAC inhibitors downregulate VLA-4 surface levels in HSC and AML cells [42], and selective HDAC6 inhibition disrupts VLA-4 mediated adhesion in ALL [43]. Together with evidence that HDAC6 regulates vesicular transport in immune cells [44, 45], these data support a model in which HDAC6 modulates integrin and chemokine receptor through microtubule-dependent vesicle transport. This may influence B-ALL cell adhesion and positioning within BM niches, although further studies are needed.

We also observed higher HDAC6 levels in BM-derived blasts compared with PB-derived blasts. Crosstalk between leukaemic cells and MSCs promotes leukaemia progression through direct contact and secretion of extracellular matrix molecules, cytokines and chemokines [6]. In mantle cell lymphoma (MCL), stromal adhesion induces c-Myc activation and miR-548m downregulation, leading to HDAC6 upregulation [46]. A similar stromal-driven mechanism may operate in B-ALL, promoting the emergence and maintenance of HDAC6^high^ clones within the BM niche.

Using a xenotransplantation model, we demonstrate that TubA treatment significantly reduces leukaemic homing to the BM and infiltration of extramedullary organs. As these processes are central determinants of relapse and disease progression [47], our findings support HDAC6 inhibition as strategy to limit leukaemia dissemination. Selective HDAC6 inhibition may offer a more targeted approach with improved tolerability compared with pan-HDAC inhibitors [48]. Future studies should evaluate whether HDAC6 inhibitors are most effective as monotherapy or in combinations with existing agents, particularly in patients with HDAC6^high^ disease.

This study has limitations. Mechanistic analyses were primarily performed in REH cells, although HDAC6 overexpression was also observed in RS4:11 cells and in primary samples from paediatric and adult cohorts. Some experiments using patient-derived material were constrained by sample availability, but consistent effects were observed across donors. While most assays used pharmacologic inhibition, key phenotypes were validated by genetic HDAC6 depletion, supporting target specificity. HDAC6 inhibition reduced leukaemic dissemination in vivo, but did not significantly extend survival, suggesting that HDAC6 targeting may be most effective in combination strategies. Finally, we were unable to perform xenograft assays using HDAC6-depleted cells due to insufficient cell numbers. To our knowledge, prior in vivo studies in ALL have also relied mainly on pharmacologic HDAC6 inhibition [43, 49]. Future xenograft studies will be essential to establish the causal contribution of HDAC6 to leukaemia dissemination in vivo.

In summary, genetic and pharmacologic inhibition across complementary functional assays identifies HDAC6 as a key regulator of B-ALL cell migration, BM homing, and organ infiltration. The highest HDAC6 levels occur in relapsed patients, underscoring its clinical relevance. These findings nominate HDAC6 as a biomarker of relapse risk and therapeutic target to limit leukaemic dissemination and improve outcomes in high-risk B-ALL.

## Supplementary information

Supplementary Material

Clinical data of paediatric B-ALL patients analyzed by RNA-seq

Correlation analysis of HDAC6 RPKM levels analyzed by RNA-seq with clinical parameters of the B-ALL patients

Clinical data of paediatric B-ALL patients analysed by flow cytometry

Correlation analysis of HDAC6 protein levels analyzed by flow cytometry with clinical parameters of the B-ALL patients

Clinical data of adult B-ALL patients included in this study

## Data Availability

All data related to this manuscript are included in the main text and supplement.
