# Supplementary material for: HDAC6 is a biomarker of leukaemic dissemination and relapse in B-ALL
Source: Br J Cancer. 2026 Jul 26;135(9):1470–82. doi: 10.1038/s41416-026-03539-2 (PMC13675229; doi:10.1038/s41416-026-03539-2)
Supplement: Supplementary file 1 [file 41416_2026_3539_MOESM1_ESM.pdf]

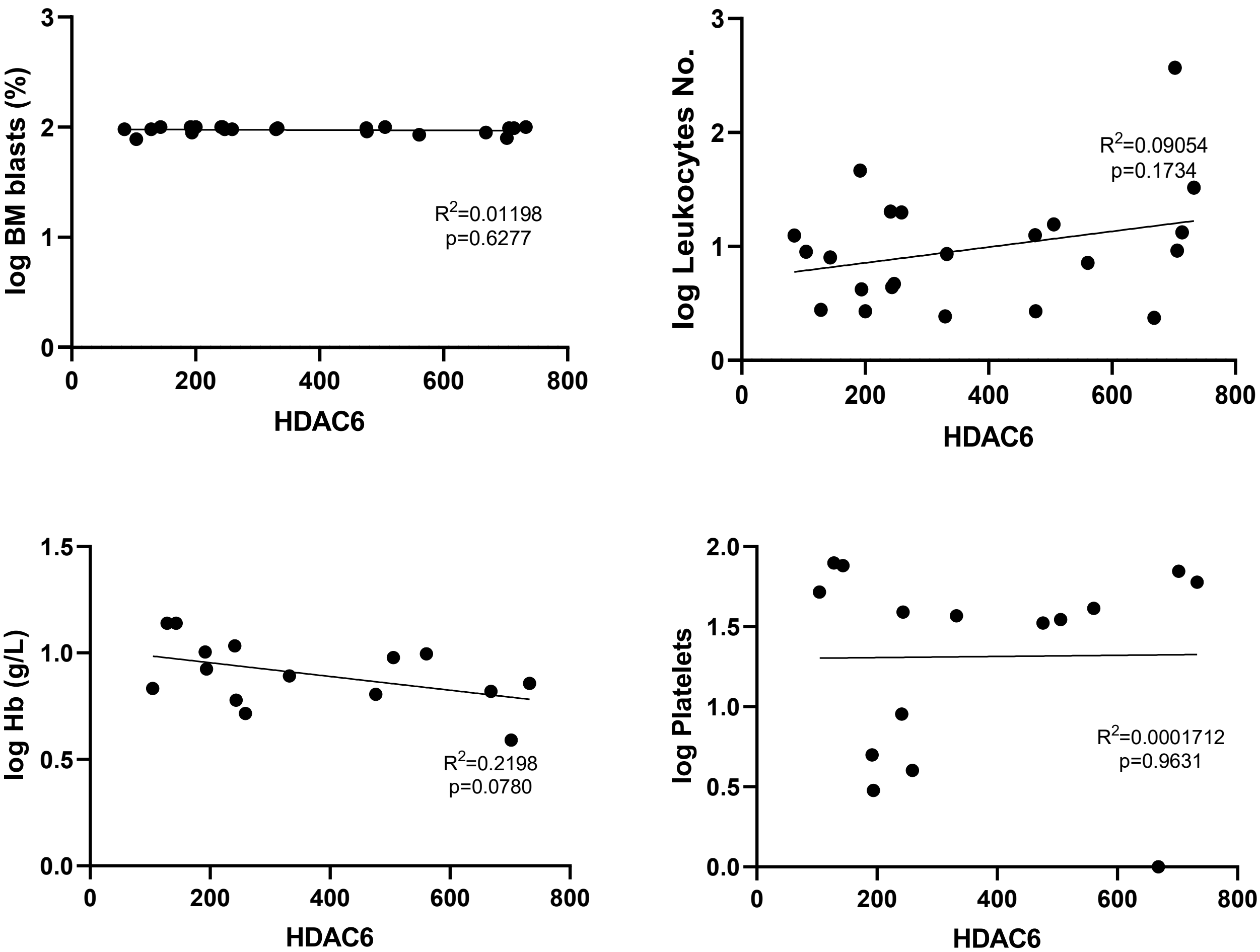

Supplementary Figure 1.

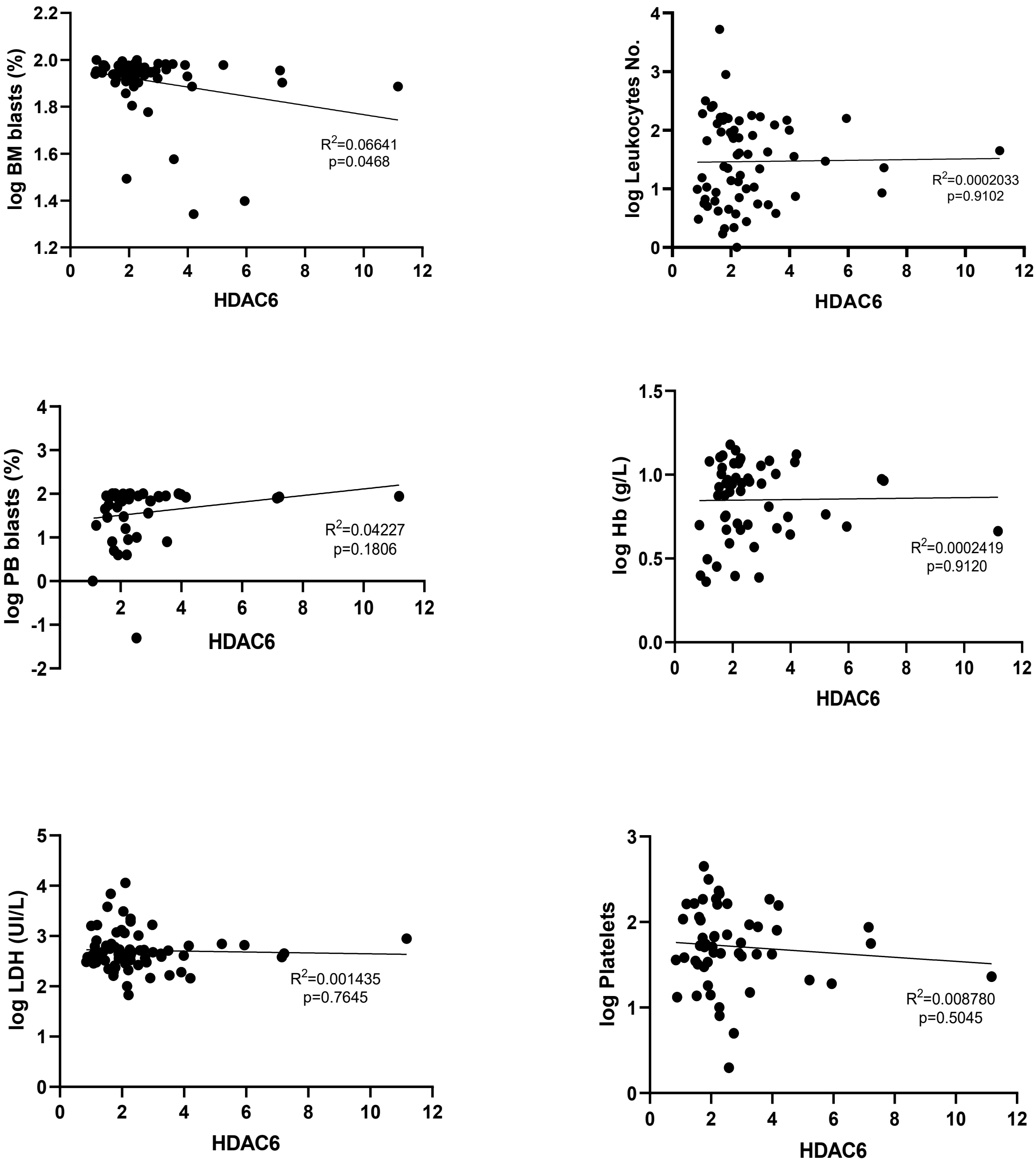

Supplementary Figure 2.

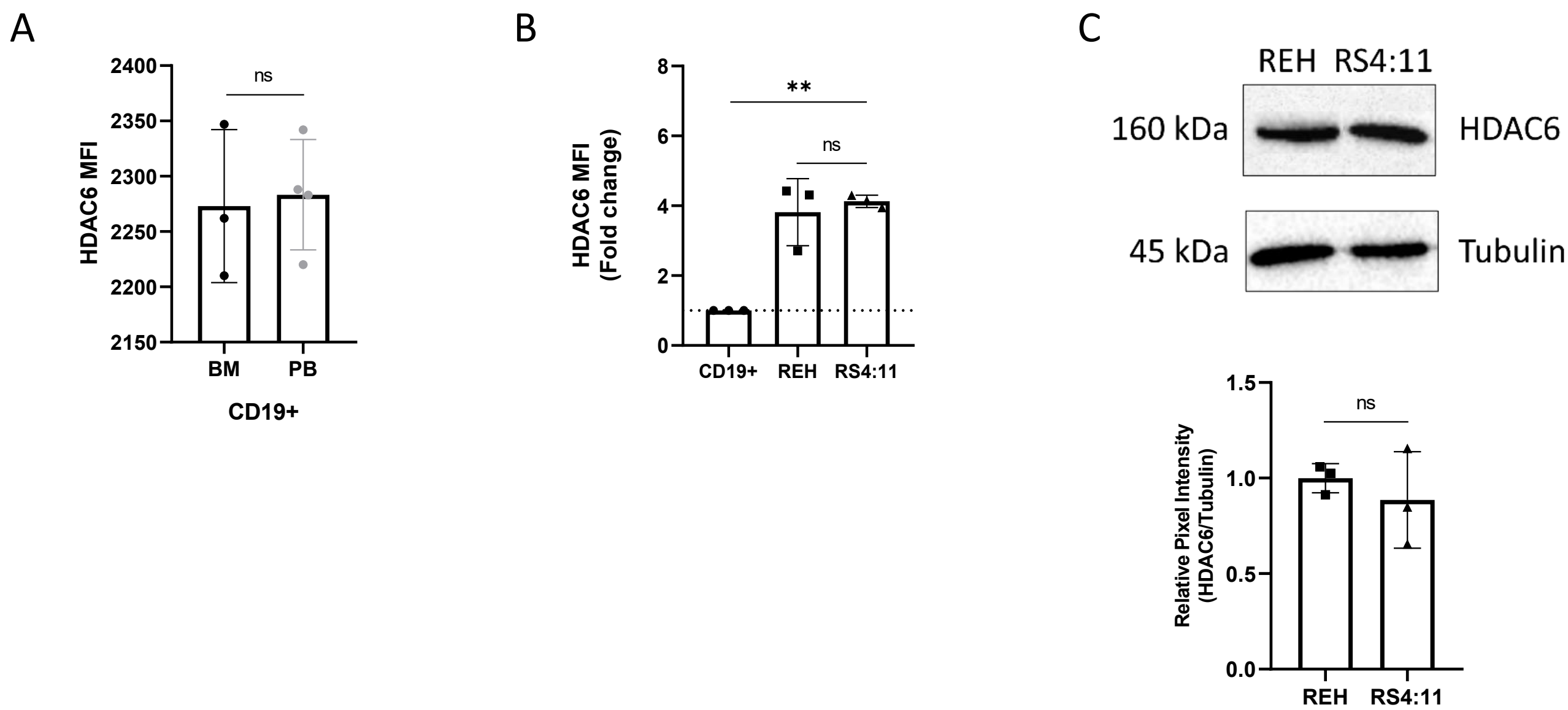

Supplementary Figure 3.

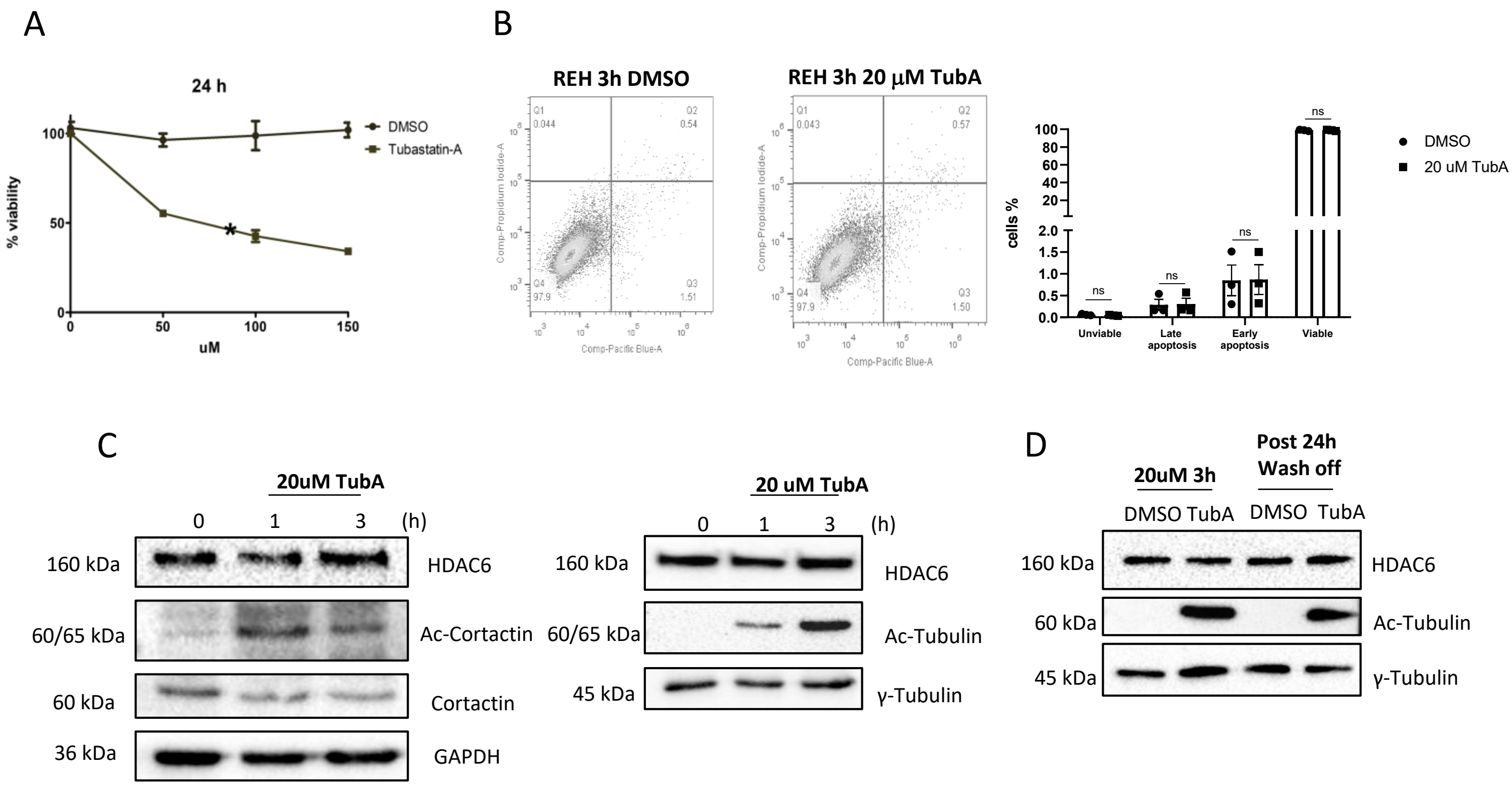

Supplementary Figure 4.

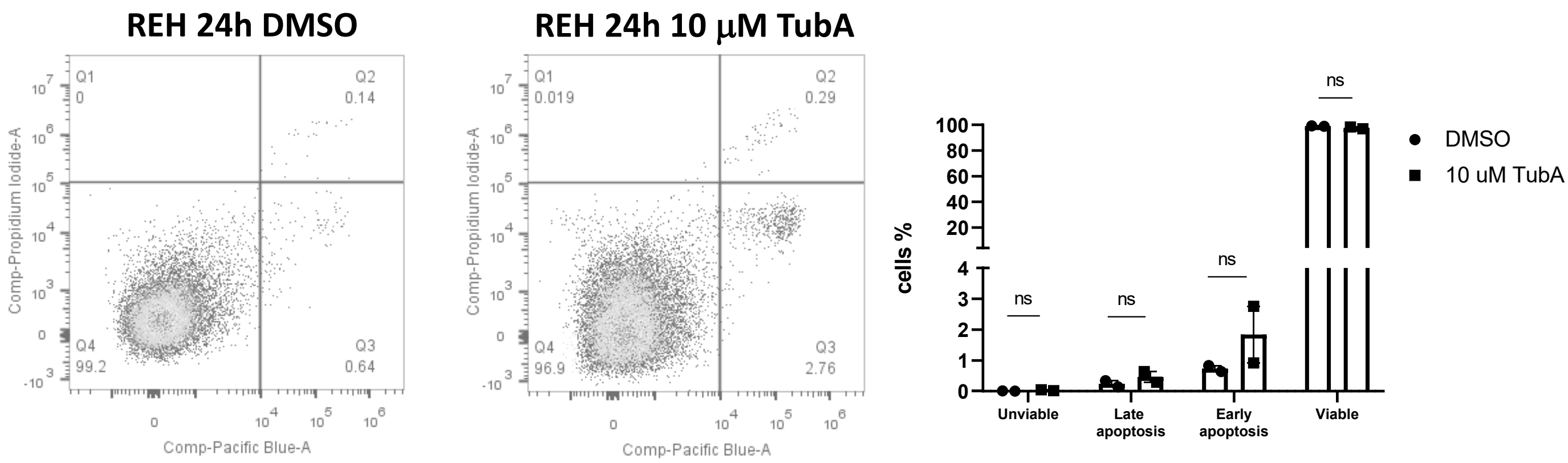

Supplementary Figure 5.

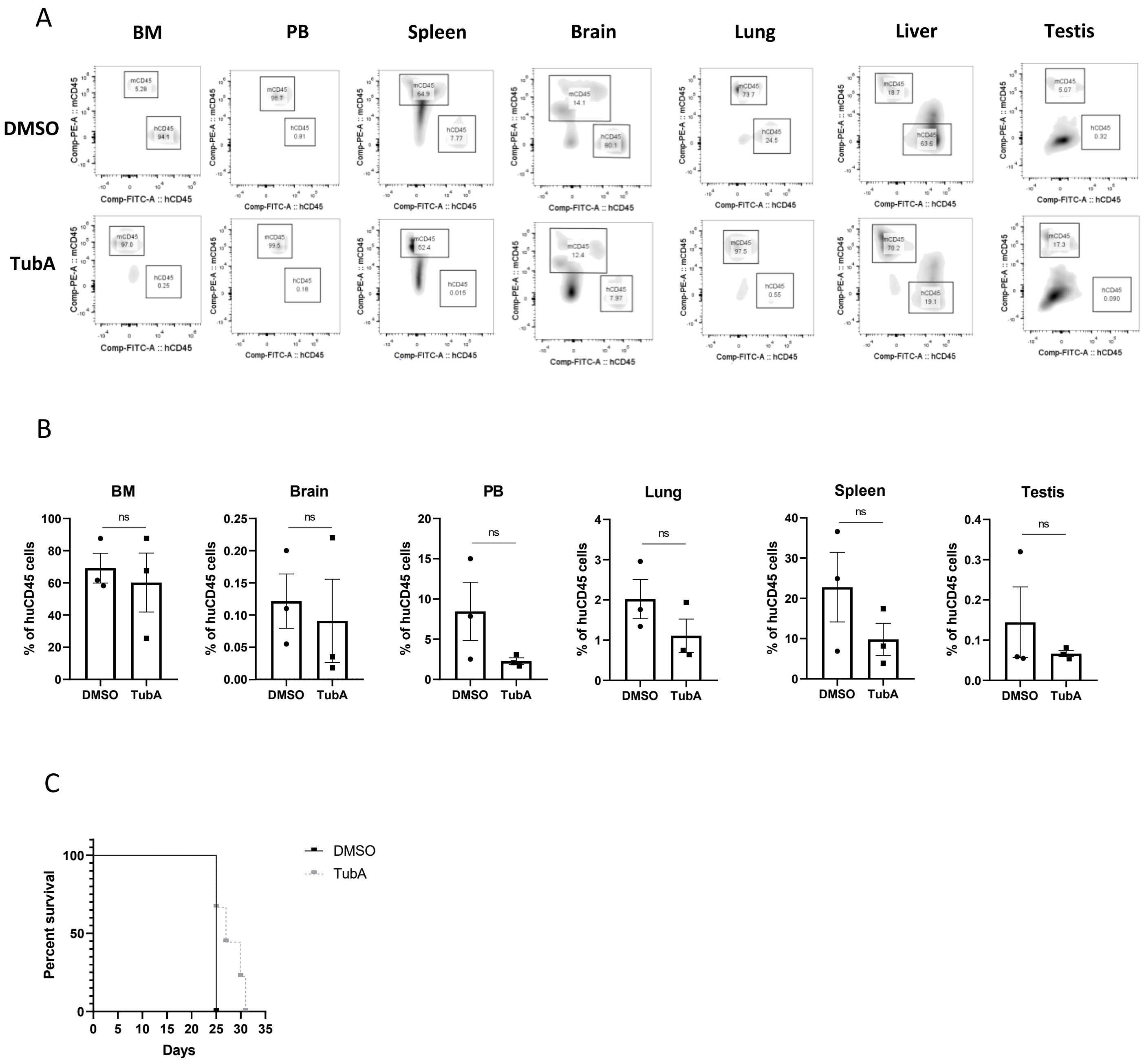

Supplementary Figure 6.

Supplementary Methods.

Flow cytometry

Mononuclear cells from ALL-BM, ALL-PB, and healthy donors were stained with FITC-anti-human CD34 (BioLegend, #304058) and PE-anti-human CD19 (BioLegend, #302254). Intracellular HDAC6 was detected following fixation and permeabilization (Perm/Wash) using Alexa Fluor™ 647-conjugated anti-HDAC6 (Santa Cruz Biotechnology). Apoptosis was assessed using the Pacific Blue™ Annexin V Apoptosis Detection Kit with propidium iodide (BioLegend).

To evaluate F-actin dynamics, REH cells untreated or treated with 20 µM TubA for 3 h were stimulated with 100 ng/mL CXCL12 for 15, 30, 60, 180 and 360 seconds. Cells were directly transferred to 4% paraformaldehyde (PFA) and incubated for 20 min at room temperature (RT). Cells were permeabilized with PermWash and Alexa Fluor™488-Phalloidin was used to stain F-actin for 30 min at RT. REH cells were stained using the following fluorochrome-coupled antibodies (Biolegend): anti-hCD49d-PE/Cy7 (#304303), anti-hCD29-APC (#303007), and anti-hCXCR4-APC (#306510). Data were acquired on a FACSCanto II (BD Biosciences) or Cytek™ Aurora cytometers and analyzed using FlowJo v10 (BD Life Sciences).

RNA-seq Libraries and Sequencing

Total RNA was extracted from BM aspirate cells from patients diagnosed with B-ALL (Suppl. Table 1) using direct-zol RNA (Zymo Research, Irvine, CA) and the libraries were constructed using TruSeq Stranded Total RNA with Ribo-Zero Gold (Illumina, San Diego, CA) as previously described (20). Libraries were sequenced for 2x75 cycles (paired-end sequencing) using an Illumina sequencing platform (NextSeq500, Illumina, San Diego, CA). The generated RNA-seq data are available at the NCBI BioProject database: https://www.ncbi.nlm.nih.gov/bioproject/?term=PRJNA809691. Data were assessed and pre-processed using FastQC v0.11.5. For trimming and adapter removal we used Trimmomatic-0.36. Then, the RNA sequence files were aligned to the human genome reference GRCh37 using Star 2.7.10b with default parameters. Quantification of transcripts was realized using Salmon (v1.10.2).

Immunofluorescence microscopy

REH cells were adhered to poly-L-lysine–coated slides, fixed with 4% paraformaldehyde, permeabilized with 0.1% Triton X-100, and blocked with 5% BSA in PBS for 1 h, followed by incubation with primary antibodies recognizing HDAC6 and cortactin overnight at 4 °C. Cells were washed and incubated with Alexa-Fluor-labelled species-specific secondary antibodies and mounted in ProLong GOLD (Thermo Fisher) containing DAPI. Images were acquired using a TCS-SP8 confocal microscope (Leica, Deer Park, IL). Confocal z-stack images were analyzed for surface-to-surface colocalization of cortactin and HDAC6 by constructing 3D surfaces and generating a new colocalized channel to identify the true colocalized regions using the masked function in Imaris software (Version 10.2, Oxford Instruments). Automatic thresholding was applied to remove background noise and accurately identify true voxels corresponding to colocalized cortactin-HDAC6 signal, as well as total cortactin and total HDAC6 signals for each cell.

Chemotaxis assay

Patient-derived B-ALL cells (1.5×10^5) from BM or PB were placed in the upper chamber of 5-µm pore Transwells. RPMI-1640 containing CXCL12 (100 ng/mL) was added to the lower chamber. After 4 h at 37°C, migrated cells were recovered and counted by trypan blue exclusion. Experiments were performed in duplicate or triplicate depending on cell availability.

Western blotting

B-ALL cells were lysed in SDS buffer (25mM HEPES pH 7.5, 2 mM EDTA, 25mM NaF, 1% SDS and complete protease inhibitor cocktail) and analyzed by standard protocols using different antibodies: anti-cortactin (clone 289H10, kindly provided by Dr. Klemens Rottner (Department of Cell Biology, Helmholtz Centre for Infection Research, Germany), anti-HDAC6 (CST, Danvers, MA, #4569), anti-acetyl-cortactin (Millipore, Temecula, California #09-881), anti-acetyl-tubulin (CST, #5335), anti-γ tubulin (ThermoScientific, Waltham, MA, #MA1-850), and anti-GAPDH (Santa Cruz, #47724).

Statistics

Statistical analyses were performed using Prism v8.0.1 (GraphPad). Two-group comparisons were analyzed using two-tailed Student's t-tests. Comparisons among multiple groups were performed using one-way or two-way ANOVA with appropriate post hoc tests. Associations between HDAC6 expression and clinical parameters were analyzed using the Mann–Whitney U test. Correlations between HDAC6 expression and continuous clinical variables were assessed using Pearson's correlation coefficient. P values < 0.05 were considered statistically significant.

Supplementary Legends.

Figure S1. Correlation of HDAC6 mRNA expression with clinical parameters in pediatric B-ALL. HDAC6 mRNA RPKM (reads per kilobase per million) values in bone marrow B cells from pediatric B-ALL patients (n = 22) were correlated with continuous clinical variables. The y-axis is log-scaled. Correlations were assessed using Pearson's correlation coefficient. The y-axis is log-scaled. Pearson's correlation coefficients (R^2) and *p* values are shown for each parameter.

Figure S2. Correlation of HDAC6 protein levels with clinical parameters in pediatric B-ALL. HDAC6 protein levels in progenitor and precursor B cells from pediatric B-ALL patients (n = 72) were quantified by flow cytometry and normalized to healthy $CD19^+$ B cells (n = 7). Correlations between HDAC6 expression and continuous clinical variables were assessed using Pearson's correlation coefficient. The y axis is log scaled. Pearson's correlation coefficients (R^2) and p values are shown for each parameter.

Figure S3. HDAC6 protein levels in healthy B cells and B-ALL cell lines.
(A) HDAC6 protein levels in $CD19^+$ B cells from healthy BM and PB. Unpaired two-tailed Student's *t* test; n = 3. **(B)** HDAC6 protein levels in REH and RS4:11 cells normalized to healthy BM $CD19^+$ B cells (set to 1, dotted line). One-way ANOVA; n = 3. **(C)** Representative immunoblots and densitometric quantification of HDAC6 in REH and RS4:11 cells. γ-Tubulin served as loading control; n = 3. Error bars indicate standard deviation. $^{**}p < 0.01$; ns, not significant.

Figure S4. TubA does not induce apoptosis at migration-inhibitory concentrations and increases in acetyl-cortactin and acetyl-tubulin levels in REH cells. (A) MTT assay of REH cell viability after 24 h exposure to TubA (50–150 µM). IC_{50} = 94 µM; n = 4. **(B)** Annexin V/PI apoptosis analysis of REH cells treated with DMSO or TubA (20 µM, 3 h). Two-way ANOVA; n = 3. **(C)** Representative immunoblots of HDAC6, acetyl-cortactin, cortactin, and acetyl-tubulin after TubA treatment (20 µM, 3 h). GAPDH and γ-tubulin served as loading controls; n = 3. **(D)** Sustained acetyl-tubulin levels after TubA washout for at least 24 h; n = 3. Error bars indicate SD; ns, not significant.

Figure S5. Low-dose TubA induces minimal apoptosis in REH cells.
Annexin V/PI analysis of REH cells treated with TubA (10 µM, 24 h) or DMSO. Two-way ANOVA; n = 2. Error bars indicate standard deviation; ns, not significant.

Figure S6. TubA reduces organ infiltration by REH cells *in vivo*.
(A) Representative flow cytometry plots of REH cells infiltrating the indicated organs of DMSO- and TubA-treated mice. Percentages of $hCD45^+$ versus $mCD45^+$ cells within the live cell population are shown. **(B)** Quantification of $hCD45^+$ leukemic cells in organs from an independent xenograft experiment in which treatment was initiated only after disease was established. Unpaired two-tailed Student's *t* test; n=3. **(C)** Kaplan–Meier survival analysis of REH-xenografted NSG mice treated with TubA or DMSO as in A. A non-significant trend to better survival was observed. Error bars indicate SEM; ns, not significant.

Supplementary Legends.

Supplementary Table 1. Clinical and biological characteristics of pediatric B-ALL patients analyzed by RNA-sequencing (n = 22). Statistical analyses are presented in Supplementary Table 2. Abbreviations: P=patient; M=months; Y=years; M=male; F=female; ND=not determined; NA=not available; BM= bone marrow; PB=peripheral blood; LDH= lactate dehydrogenase; RPKM=reads per kilobase millions.

Supplementary Table 2. Correlation analysis of HDAC6 RPKM levels obtained by RNA-seq with clinical parameters of pediatric B-ALL patients. Mann–Whitney U-test with an α of 5% was applied to define statistical significance.

Supplementary Table 3. Clinical and biological characteristics of pediatric B-ALL patients analyzed by flow cytometry (n = 72). For a subset of patients with paired debut–relapse samples (n = 16), each time point is listed as an independent biological sample. HDAC6 fold-change values measured by flow cytometry are included as part of the biological characterization of the cohort and correspond to the values used in Figure 1B–C; statistical analyses are presented in Supplementary Table 4. Abbreviations: P=patient; M=months; Y=years; M=male; F=female; ND=not determined; NA=not available; BM= bone marrow; PB=peripheral blood; LDH= lactate dehydrogenase.

Supplementary Table 4. Correlation analysis of HDAC6 protein levels measured by flow cytometry across clinical parameters of the pediatric B-ALL patients. Mann–Whitney U-test with an α of 5% was applied to define statistical significance. Significant values are highlighted in bold.

Supplementary Table 5. Clinical characteristics and HDAC6 protein levels of adult B-ALL analyzed by flow cytometry. Adult B-ALL samples (n = 54) were obtained at diagnosis from patients treated at IMSS Centro Médico Nacional La Raza (Mexico City). HDAC6 expression is reported as fold change of mean fluorescence intensity relative to healthy $CD19^{+}$ B-cell controls. Abbreviations: P=patient; Y=years; M=male; F=female.
